# Inhibitory effect of microalgae and cyanobacteria extracts on influenza virus replication and neuraminidase activity

**DOI:** 10.7717/peerj.5716

**Published:** 2018-10-26

**Authors:** Thauane Silva, Paulo S. Salomon, Lidilhone Hamerski, Juline Walter, Rafael B. Menezes, José Edson Siqueira, Aline Santos, Jéssica Aparecida Morais Santos, Natália Ferme, Thaise Guimarães, Giovana O. Fistarol, Paulo I. Hargreaves, Cristiane Thompson, Fabiano Thompson, Thiago Moreno Souza, Marilda Siqueira, Milene Miranda

**Affiliations:** 1 Laboratório de Vírus Respiratórios e do Sarampo, Instituto Oswaldo Cruz, Fundação Oswaldo Cruz, Rio de Janeiro, Brazil; 2 Laboratório de Fitoplâncton Marinho, Instituto de Biologia, Universidade Federal do Rio de Janeiro, Rio de Janeiro, Brazil; 3 Instituto de Pesquisas de Produtos Naturais, Universidade Federal do Rio de Janeiro, Rio de Janeiro, Brazil; 4 Laboratório de Microbiologia Marinha, Instituto de Biologia, Universidade Federal do Rio de Janeiro, Rio de Janeiro, Brazil; 5 Laboratório de Imunofarmacologia, Instituto Oswaldo Cruz, Fundação Oswaldo Cruz, Rio de Janeiro, Brazil; 6 Centro de Desenvolvimento Tecnológico em Saúde, Fundação Oswaldo Cruz, Rio de Janeiro, Brazil

**Keywords:** Cyanobacteria, Microalgae, Neuraminidase inhibition, Anti-influenza extracts, OST-sensitive and resistant influenza viruses

## Abstract

**Background:**

The influenza virus can cause seasonal infections with mild to severe symptoms, circulating worldwide, and it can affect people in any age group. Therefore, this infection is a serious public health problem that causes severe illness and death in high-risk populations. Every year, 0.5% of the world’s population is infected by this pathogen. This percentage can increase up to ten times during pandemics. Influenza vaccination is the most effective way to prevent disease. In addition, anti-influenza drugs are essential for prophylactic and therapeutic interventions. The oseltamivir (OST, a neuraminidase inhibitor) is the primary antiviral used in clinics during outbreaks. However, OST resistant viruses may emerge naturally or due to antiviral pressure, with a prevalence of 1–2% worldwide. Thus, the search for new anti-influenza drugs is extremely important. Currently, several groups have been developing studies describing the biotechnological potential of microalgae and cyanobacteria, including antiviral activity of their extracts. In Brazil, this potential is poorly known and explored.

**Methods:**

With the aim of increasing the knowledge on this topic, 38 extracts from microalgae and cyanobacteria isolated from marine and freshwater biomes in Brazil were tested against: cellular toxicity; OST-sensitive and resistant influenza replications; and neuraminidase activity.

**Results:**

For this purpose, Madin-Darby Canine Kidney (MDCK)-infected cells were treated with 200 μg/mL of each extract. A total of 17 extracts (45%) inhibited influenza A replication, with seven of them resulting in more than 80% inhibition. Moreover, functional assays performed with viral neuraminidase revealed two extracts (from *Leptolyngbya* sp. and Chlorellaceae) with IC_50_ mean < 210 μg/mL for influenza A and B, and also OST-sensitive and resistant strains. Furthermore, MDCK cells exposed to 1 mg/mL of all the extracts showed viability higher than 80%.

**Discussion:**

Our results suggest that extracts of microalgae and cyanobacteria have promising anti-influenza properties. Further chemical investigation should be conducted to isolate the active compounds for the development of new anti-influenza drugs. The data generated contribute to the knowledge of the biotechnological potential of Brazilian biomes that are still little explored for this purpose.

## Introduction

Lower acute respiratory infections (ARIs) are a persistent and pervasive public health problem, since they constitute one of the main causes of morbidity and mortality, with greater burden of disease worldwide than human immunodeficiency virus infection, malaria, cancer, or heart attacks (Mizgerd, 2008; Pichon, Lina & Josset, 2017).

World Health Organization (WHO) data indicate influenza A viruses as the main viral agents causing ARI, and therefore of great epidemiological importance. Seasonal influenza epidemics, currently involving influenza A(H3N2), A(H1N1)pdm09 and B (Yamagata (Yam) and Victoria (Vic) lineages), affect 10–20% of the human population each year (WHO, 2018b). According to new estimates by the United States Centers for Disease Control and Prevention, up to 650,000 deaths annually are associated with respiratory diseases from seasonal influenza (WHO, 2017). Although influenza B only infects humans, influenza A infects both humans and animals. Furthermore, humans can be infected with avian, swine and other zoonotic influenza viruses, such as A(H5N1), A(H7N9), and A(H9N2) subtypes (WHO, 2018a).

The influenza A virus belongs to *Orthomyxoviridae* family. The genomic single stranded RNA (RNAss) is octa-segmented, negative-sense, surrounded by a helical capsid with externally lipoprotein envelope, in which glycoproteins hemagglutinin (HA) and neuraminidase (NA) are inserted (Camp et al., 2013). Frequently, minor modifications in these envelope proteins may alter the affinity of vaccine antibodies or inactivate them, preventing recognition of the virus by the immune system, causing repetitive influenza outbreaks worldwide. However, in rare moments the combination of the eight genomic segments (reassortment) can occur, such as between influenza animal and human subtypes. This event, named antigenic shift, can result in strains capable of causing large regional or global pandemic outbreaks (Zhu, Wang & Wang, 2017).

The primary method of prevention is annual vaccination. Antiviral medications for prevention and treatment of influenza are an important adjunct to vaccines, especially for at-risk groups, including young children, older people, pregnant women and people with certain health conditions (Del Giudice & Rappuoli, 2015; Rotrosen & Neuzil, 2017).

The most important class of antiviral recommended for the control of influenza epidemics and eventual pandemics is the Neuraminidase Inhibitors (NAIs), particularly oseltamivir (OST) and zanamivir (ZAN). These compounds are active against all influenza A subtypes and the two major influenza B lineages. Thus, the emergence of NAIs resistance could be a major clinical concern. Although most currently circulating influenza A and B strains are susceptible to NAIs, the pressure imposed by OST has led to the selection of OST-resistant mutants, with a prevalence of 1–2% in different countries (Dixit et al., 2013; Lopes e Souza et al., 2015; Souza et al., 2011). The OST-resistant strains with compensatory mutations may arise in an independent fashion, with samples being identified in different states of Brazil and in other countries (Lopes e Souza et al., 2015). Reports have shown single or multiple substitutions or deletions in the NA gene, which can promote a phenotype cross-resistance to the two main NAIs (oseltamivir and zanamivir) used in clinics, mostly in immunocompromised individuals (Abed & Boivin, 2017). Systematic circulation of these viral strains may jeopardise the use of the first line of anti-influenza drugs in the future. Thus, the search for new anti-influenza compounds is pivotal for public health.

Marine and freshwater natural products have been reported to contain different biological activities, which allow them to provide health and cosmetic benefits, such as antioxidant, anti-inflammatory, anticancer and antiviral activity, including against some respiratory viruses (Chen et al., 2016; Hasui et al., 1995; Khalid et al., 2017; Mayer & Hamann, 2004; Mayer et al., 2007; Mayer et al., 2017; Mendes Gda et al., 2010). Microalgae and cyanobacteria are huge natural sources of high-value compounds with health-promoting properties. These marine and freshwater organisms are a rich source of bioactive compounds such as vitamins, proteins with essential amino acids, polysaccharides, fatty acids, minerals, enzymes, fiber and photosynthetic pigments (carotenoids and chlorophylls) (Montalvao et al., 2016). The carotenoids contain a great compound range of different classes that are biosynthesized by condensation of isoprene units in (micro)algae and cyanobacteria (Gong & Bassi, 2016; Hynstova et al., 2018; Sathasivam & Ki, 2018).

The aim of this study is to evaluate the antiviral activity of natural extracts against influenza A and B, oseltamivir-sensitive and resistant strains and lineages. The 38 extracts were obtained from microalgae and cyanobacteria cultures isolated from fresh (Paraguaçu River and Lençois Maranhenses Lakes) and marine water (Abrolhos Bank and Guanabara Bay) biomes in Brazil. We evaluated these extracts for cellular toxicity, influenza replication and neuraminidase activity. The extracts presented low cytotoxicity, and 17 of them inhibited influenza replication in more than 80% *in vitro*, with two inhibiting neuraminidase activity from influenza A and B OST-sensitive and resistant. These data contribute to the knowledge of the biotechnological potential of Brazilian biomes, which are underexplored for antiviral research and bioprospecting.

## Materials and Methods

### Origin of microalgae and cyanobacteria strains

Microalgae and cyanobacteria strains used in this study belong to the Culture Collection of Microalgae at UFRJ (CCMR, Marine Phytoplankton Laboratory, Federal University of Rio de Janeiro). These strains were isolated by single-cell sorting in a flow cytometer (Fistarol et al., 2018) and are maintained in culture by successive transfers to fresh liquid medium at 3–6 week intervals, depending on the species. All 26 strains used here are originated from fresh (Paraguaçu River and Shallow Lake System of Lençois Maranhenses) and marine water (Guanabara Bay estuary and Abrolhos coral reefs) biomes in Brazil (Table 1). Taxonomic identification of the strains was based on morphology and, for most of them, also on DNA sequencing (SSU rDNA) (Fistarol et al., 2018). The field experiments were approved by Ministério do Meio Ambiente (MMA), Instituto Chico Mendes de Conservação da Biodiversidade (ICMBio) and Sistema de Autorização e Informação em Biodiversidade (SISBIO), approval number: 35854-2. The access to genetic heritage was approved by Conselho Nacional de Desenvolvimento Científico e Tecnológico (CNPq), approval number: 010339/2014-0; and Sistema Nacional de Gestão do Patrimônio Genético e do Conhecimento Tradicional Associado (SisGen), approval number: A03D5C4.

**Table 1:** Extracts identification and collection specifications.

**Extract identification**	**Taxon**	**Source**	**Collection point**	**Latitude**	**Longitude**	**Collection data**
1	Unidentified filamentous green algae	Freshwater	Lençois Maranhenses Lakes	2°32′11″S	41°51′10″W	August, 2012
2	*Staurastrum* sp.	Freshwater	Lençois Maranhenses Lakes	2°30′10″S	42°51′50″W	August, 2012
3	Unidentified filamentous green algae	Freshwater	Paraguaçu River	12°57′8.48″S	41°16′39.29″W	November, 2012
4	*Scenedesmus* sp.	Freshwater	Lençois Maranhenses Lakes	12°50′25.91″S	41°19′26.52″W	November, 2012
5	*Leptolyngbya* sp.^a^	Marine	Abrolhos Bank	17°59′52.8″S	38°40′15.6″W	October, 2013
6	Unidentified coccoid green algae	Freshwater	Paraguaçu River	12°57′8.48″S	41°16′39.29″W	November, 2012
7	Unidentified coccoid green algae	Freshwater	Lençois Maranhenses Lakes	12°50′25.91″S	41°19′26.52″W	November, 2012
8	*Scenedesmus abundans*	Freshwater	Lençois Maranhenses Lakes	2°32′11″S	41°51′10″W	August, 2012
9	*Scenedesmus vacuolatus*	Freshwater	Lençois Maranhenses Lakes	2°30′10″S	42°51′50″W	August, 2012
10	*Chlamydomonas angulosa*	Freshwater	Lençois Maranhenses Lakes	2°38′50″S	42°49′43″W	August, 2012
11	Chlorellaceae	Freshwater	Lençois Maranhenses Lakes	2°38′50″S	42°49′43″W	August, 2012
12	Desmidiaceae	Freshwater	Lençois Maranhenses Lakes	2°35′08″S	42°48′03″W	August, 2012
13	*Chlamydomonas sp.*	Freshwater	Lençois Maranhenses Lakes	2°30′10″S	42°51′50″W	August, 2012
14	*Selenastrum* sp.	Freshwater	Lençois Maranhenses Lakes	2°30′10″S	42°51′50″W	August, 2012
15	Scenedesmaceae	Freshwater	Lençois Maranhenses Lakes	2°33′42″S	42°51′49″W	August, 2012
16	Unidentified coccoid green algae	Freshwater	Lençois Maranhenses Lakes	2°30′10″S	42°51′50″W	August, 2012
17	*Leptolyngbya* sp.^a^	Marine	Abrolhos Bank	12°50′25.91″S	41°19′26.52″W	November, 2012
18	*Desmodesmus armatus*	Freshwater	Lençois Maranhenses Lakes	2°30′10″S	42°51′50″W	August, 2012
19	*Symbiodinium* sp.	Marine	Abrolhos Bank	17°57′32.7″S	38°30′20.3″W	March, 2012
20	*Chattonella sp.*	Marine	Guanabara Bay	22°50′01.0″S	43°12′29.0″W	January, 2016
21	Chlorellaceae	Freshwater	Lençois Maranhenses Lakes	2°30′10″S	42°51′50″W	August, 2012
22	*Desmodesmus perforatus*	Freshwater	Lençois Maranhenses Lakes	2°30′10″S	42°51′50″W	August, 2012
23	Chlorellaceae	Freshwater	Lençois Maranhenses Lakes	2°32′11″S	41°51′10″W	August, 2012
24	Desmidiaceae	Freshwater	Lençois Maranhenses Lakes	2°30′10″S	42°51′50″W	August, 2012
25	*Romeria* sp.^a^	Marine	Abrolhos Bank	18°00′13.0″S	39°14′57.0″W	February, 2014
26	*Nanofrustulum shiloi*	Marine	Abrolhos Bank	17°57′07.0″S	39°13′11.0″W	February, 2013

**Notes.**

a Cyanobacteria strains.

### Microalgae and cyanobacteria cultivation

Microalgae and cyanobacteria were grown in autoclaved sterile medium at 26 ± 1 °C, with a photon flux of ca. 300 µmoles photon m^−2^ s^−1^ and 16 h light: 8 h dark photoperiod. Freshwater strains were grown in ASM-1 medium whereas marine strains were grown in f/2 medium (Gorham et al., 1964; Guillard & Lorenzen, 1972; Reynolds & Jaworski, 1978). Cultures were grown in glass balloons with 300–500 mL of culture medium for 3–6 weeks, depending on the growth rate of each strain. The cultures were stored at −80 °C until extracts production.

### Extracts production

All the microalgae and cyanobacteria cultures (cells and culture medium) were frozen, thawed and filtered through cheesecloth. The filtrate was extracted three times with ethyl acetate (added ratio 1:1 of volume) and three times with n-butanol (added ratio 1:1 of volume). The organic layers were concentrated under reduced pressure to dryness. A total of 38 extracts were produced, 26 in ethyl acetate (EtOAc) and 12 in n-butanol (n-BuOH). The crude extracts were stored at −20 °C until analysis. The extracts from one to 12 strains were obtained in the first batch with both solvents. After the screening test for anti-influenza activity, the second batch of extracts was produced from 13 to 26 strains, and they were solely obtained with ethyl acetate. Dried extracts were resuspended in dimethyl sulfoxide (DMSO) in a final concentration of 100 mg/mL for the *in vitro* tests. All the extracts were analyzed by TLC, HPLC, and Mass Spectrometry (MS). However, we could not identify any compounds, but the chemical profiles of the active extracts were preserved. Thus, the procedure must be repeated on a larger scale for the more active anti-influenza extracts. The chromatograms and mass spectra for extracts 5 and 21 are provided in the File S1 (*S01*).

### Cells and virus

Madin-Darby canine kidney (MDCK) cells (London line) were kindly donated by the Centers for Disease Control and Prevention (CDC), Influenza Reagent Resources (IRR) (FR-58). These cells were used for cytotoxicity analysis, viral growth and experimental assays with influenza virus infection. These cells were cultured in Dulbecco’s modified Eagle’s medium (DMEM; Gibco, Waltham, MA, USA) supplemented with 10% of *fetal bovine serum (FBS;* Gibco) and 100 U/mL penicillin and 100 mg/mL streptomycin (Sigma-Aldrich, St. Louis, MO, USA). Cells were cultured at 37 °C in 5% CO_2_ atmosphere (Szretter, Balish & Katz, 2006).

The viruses were grown and titrated according to WHO manual for the laboratory diagnosis and virological surveillance of influenza (WHO, 2011). The virus titration was performed by 50% Tissue Culture Infectious Dose (TCID_50_) assay. We analyzed the inhibitory effect of the extracts against influenza A strains and B lineages, OST-sensitive (Wild type—WT) and resistant (mutant): A/California/04/2009 (A(H1N1)pdm09-WT); A/Perth/261/2009 (A(H1N1)pdm09-H275Y); A/Switzerland/9715293/2013 (A(H3N2)-WT); B/Phuket/3073/2013 Yam (WT); B/Brisbane/60/2008 Vic (WT); B/Perth/211/2001 Yam (WT) and B/Perth/211/2001 Yam (D197E). All the viruses were kindly provided by the International Society for Influenza and other Respiratory Viruses Diseases-Antiviral Group (isirv-AVG) and CDC –IRR.

### Cytotoxicity assay

One hundred microliters of 2.0 × 10^4^ MDCK cells were seeded into the 96-well culture plates (flat bottom) and grown for 24 h at 37 °C in a 5% CO_2_ atmosphere, then, the extracts were added with a final concentration of 1 mg/mL in DMEM. Control cells were treated with 1.0% DMSO, which did not affect the growth of the cells. The extracts were diluted in culture medium DMEM, the DMSO final concentration was equal to 1.0% (v/v). After 48 h of cells incubation with extracts or vehicle (DMSO), a freshly prepared XTT (Sigma) solution was added, as specified by the manufacturer’s instructions. The XTT is a colorless or slightly yellow compound that is reduced to formazan (bright orange) by mitochondrial dehydrogenases of viable cells, as described before (Roehm et al., 1991; Scudiero et al., 1988). The cell viability was calculated by the comparison between absorbance (A475 nm–A660 nm) from treated (A) and untreated (B) cells using the formula: A/B × 100. Tests were carried out performing three replicates (*n* = 3).

### Screening of extracts on viral replication

Initially, we performed a screening to evaluate the inhibitory effect of the extracts. MDCK cells (2.0 × 10^5^ cells/well) were seeded in 24-well tissue culture plates and incubated overnight. Then, the monolayers of cells were infected with A(H1N1)pdm09-WT or A(H3N2)-WT at 400 TCID_50_ per well during 1 h at 37 °C and 5% CO_2_. After, the viral suspension was removed and the monolayers were treated with 200 µg/mL of each extract. There is no established protocol or concentration for the anti-influenza screening of crude extracts of natural products. There are papers that use 2 mg/ml as the maximum concentration, while others use 100 µg/ml (Ding et al., 2017; Ghoke et al., 2018; Rajasekaran et al., 2013; Shoji et al., 2017; Sood et al., 2012). At 48 h post-infection (hpi), the supernatant was harvested to quantify the influenza titer. For this purpose, the supernatants were diluted 1:5 in NA-Star buffer and the neuraminidase activity was analyzed in triplicate by the NA-StarTM assay kit (Life Technologies, Carlsbad, CA, USA), according to the manufacturer’s instructions. The antiviral activity of the extracts was calculated with respect to virus control only.

### EC_50_ determination

Half maximal effective concentration was determined for the extract that inhibited influenza replication in more than 80%. MDCK cells were seeded and infected as described above. After 1 hpi, the viral suspension was removed and the monolayers were treated with different extract concentrations (400 − 12.5 µg/mL). Oseltamivir carboxylate (100 − 3.2 µM) (OST-car; kindly donated by Hoffman-La Roche Inc., Basel, Switzerland) was used as a control. For influenza titration, the supernatants were harvested at 48 hpi, and the influenza virus was titrated by TCID50/mL using MDCK cells (5 × 104 cells/well in 96-well plates) through the Reed and Muench method (Reed & Mounch, 1938; Sacramento et al., 2015). Non-linear regression of the dose response curves was performed to determine the 50% inhibitory effect on viral replication (EC_50_) for the extracts and reference compound (OST-car).

### Functional antiviral assay—IC_50_ determination

For extracts with high influenza inhibition (80%) in the screening test, we performed a functional antiviral assay to determine the concentration required to inhibit 50% of the NA enzymatic activity (IC_50_). Wild-type and resistant strains of influenza A and B were tested against different concentrations of extracts (400 − 12.5 or 200 − 6.25 µg/mL), OST-car (1,000 − 0.01 nM) was used as control. This assay was done using the NA-Star™ assay kit (Life Technologies, USA), according to the manufacturer’s instructions (Lopes e Souza et al., 2015; Souza et al., 2011; Souza et al., 2013). The IC50 was calculated using a non-linear regression.

### Statistical analysis

The dose–response curves used to calculate the pharmacological parameter values were generated using Excel 2010 for Windows software (Microsoft) (Souza et al., 2007). All of the experiments were performed at least three times, and the results are displayed as the mean or the mean ± standard error (SEM).

## Results

### Low cytotoxicity of the extracts

The cells viability was the first analysis conducted, extracts with high cell toxicity *in vitro* were disregarded from the antiviral analysis. All the extracts produced were tested at 1 mg/mL in MDCK cell cultures. As control, we exposed MDCK to DMSO in the same proportion as we used in the extracts test, 1.0% V/V in culture medium, which did not affect the cells viability. The results showed that redox mitochondrial activity of the tested cells with extracts was inhibited at most by 20% after 48 h of exposure. Approximately 60% of extracts in ethyl acetate (EtOAc) and n-butanol (n-BuOH) solvent showed 90% of cell viability (Table 2).

**Table 2:** Percentage of cell viability and influenza inhibition (screening) after exposure to extracts in ethyl acetate (EtOAc) or n-butanol (n-BuOH) solvent.

**Extract**	**Taxon**	**Cell viability (%)**		**Screening (%)**			
				**A(H1N1)pdm09-WT**		**A(H3N2)-WT**	
		**EtOAc**	**n-BuOH**	**EtOAc**	**n-BuOH**	**EtOAc**	**n-BuOH**
1	Unidentified filamentous green algae	80 ± 1	80 ± 2	87 ± 2	NI	82 ± 2	NI
2	*Staurastrum* sp.	90 ± 2	90 ± 2	85 ± 2	NI	82 ± 1	NI
3	Unidentified filamentous green algae	80 ± 1	80 ± 2	NI	30 ± 4	NI	20 ± 2
4	*Scenedesmus* sp.	90 ± 2	90 ± 2	83 ± 3	NI	80 ± 3	NI
5	*Leptolyngbya* sp.^a^	90 ± 2	90 ± 1	90 ± 2	NI	85 ± 2	NI
6	Unidentified coccoid green algae	90 ± 3	90 ± 1	73 ± 2	NI	70 ± 4	NI
7	Unidentified coccoid green algae	90 ± 1	90 ± 2	NI	NI	NI	NI
8	*Scenedesmus abundans*	90 ± 3	90 ± 2	70 ± 4	NI	75 ± 1	NI
9	*Scenedesmus vacuolatus*	80 ± 2	80 ± 2	NI	NI	NI	NI
10	*Chlamydomonas angulosa*	90 ± 1	90 ± 1	NI	NI	NI	NI
11	Chlorellaceae	80 ± 3	80 ± 4	NI	NI	NI	NI
12	Desmidiaceae	80 ± 1	80 ± 2	60 ± 2	NI	56 ± 2	NI
13	*Chlamydomonas sp.*	90 ± 3	NP	NI	NP	NI	NP
14	*Selenastrum* sp.	80 ± 2	NP	NI	NP	NI	NP
15	Scenedesmaceae	90 ± 3	NP	NI	NP	NI	NP
16	Unidentified coccoid green algae	90 ± 4	NP	NI	NP	NI	NP
17	*Leptolyngbya* sp.^a^	90 ± 3	NP	57 ± 2	NP	55 ± 2	NP
18	*Desmodesmus armatus*	80 ± 3	NP	94 ± 2	NP	90 ± 2	NP
19	*Symbiodinium* sp.	80 ± 2	NP	59 ± 4	NP	62 ± 4	NP
20	*Chattonella* sp.	90 ± 1	NP	70 ± 5	NP	65 ± 2	NP
21	Chlorellaceae	90 ± 4	NP	99 ± 1	NP	95 ± 1	NP
22	*Desmodesmus perforates*	80 ± 3	NP	58 ± 3	NP	50 ± 4	NP
23	Chlorellaceae	90 ± 3	NP	46 ± 2	NP	38 ± 4	NP
24	Desmidiaceae	90 ± 1	NP	84 ± 1	NP	82 ± 2	NP
25	*Romeria* sp.^a^	80 ± 3	NP	NI	NP	NI	NP
26	*Nanofrustulum shiloi*	90 ± 3	NP	66 ± 2	NP	60 ± 2	NP

**Notes.**

a Cyanobacteria strains.

NP not produced NI no inhibition

DMSO was used as a control at 0.01% V/V, and the cell viability remained at 100%.

### The extracts inhibit influenza replication in more than 80%

After cytotoxicity analysis, we tested anti-influenza activity with all the extracts produced, EtOAc and n-BuOH. For this purpose, MDCK-infected cells were treated with 200 µg/mL of each extract. After 48 hpi, the supernatants were harvested and the influenza neuraminidase activity was analyzed. We observed that 17 extracts (16 in EtOAc and 1 in n-BuOH) inhibited the replication of influenza A strains, A(H1N1)pdm09-WT and A(H3N2)-WT. Seven of them (41%) resulted in more than 80% of inhibition (Table 2). Just one n-BuOH extract from filamentous green algae was capable of inhibiting influenza replication, but at low levels. Despite this, an EtOAc extract obtained from another filamentous green alga was able to produce satisfactory inhibitory effects (Table 2). All the EtOAc extract from Desmidiaceae family (*Staurastrum* sp.) and four out of six EtOAc extract from Scenedesmaceae family (*Scenedesmus* sp. and *Desmodesmus* sp.) were capable of inhibiting influenza replication with different magnitudes (Table 2). Besides these, extract 21, obtained from Chlorellaceae green microalgae presented the greatest capacity to inhibit both influenza replications. However, others from the same family had limited or null capacity against this virus (Table 2).

### EC_50_ determination and SI evaluation

We determined the EC_50_ for seven marine extracts that *inhibited* in more than 80% the replication of both influenza A(H1N1)pdm09-WT and A(H3N2)-WT in the screening assay. The selective index (SI) is determined by the ratio between CC_50_ and EC_50_, and represents the relative effectiveness of the investigational product in inhibiting viral replication compared to inducing cell death. The EC_50_ of five extracts was less than 100 µg/mL with SI higher than 10.0 (Table 3). The oseltamivir carboxylate (OST-car) was used as positive control with 177,667 and 66,625 SI for A(H1N1)pdm09-WT and A(H3N2)-WT, respectively. The SI values between a pool of molecules (extracts) and a molecule chemically defined (OST) are incomparable.

**Table 3:** The EC_50_, CC_50_ and SI values from extracts that inhibited influenza A replication higher than 80% in screening assay.

**Extract**	**Taxon**	**EC**_50_^a^**(µg/mL)**		**CC**_50_^b^**(µg/mL)**	**SI**^c^	
		**A(H1N1)pdm09-WT**	**A(H3N2)-WT**		**A(H1N1)pdm09-WT**	**A(H3N2)-WT**
1	Unidentified filamentous green algae	130	150	>1,000	>7.7	>6.7
2	*Staurastrum* sp.	70	90	>1,000	>14.3	>11.1
4	*Scenedesmus* sp.	130	130	>1,000	>7.7	>7.7
5	*Leptolyngbya* sp.^d^	80	85	>1,000	>12.5	>11.8
18	*Desmodesmus armatus*	55	60	>1,000	>18.4	>16.7
21	Chlorellaceae	30	40	>1,000	>33.3	>25.0
24	Desmidiaceae	50	55	>1,000	>20.0	>18.2
OST-car		0.012 µM	0.032 µM	2,132 µM	177,7	66,63

**Notes.**

a EC_50_, the concentration required to reduced inhibition of viral infection-induced cytopathogenicity by 50%. Values represent the mean of duplicate samples from three independent experiments.

b CC_50_, the concentration required to reduced normal, non-infected cell viability by 50%. Values represent the mean of duplicate samples from three independent experiments.

c SI, selective index is determined by the ratio between CC_50_ and EC_50_.

d Cyanobacteria strain.

OST-car OST carboxylate

### Neuraminidase inhibition and IC_50_ determination

**Figure 1:**
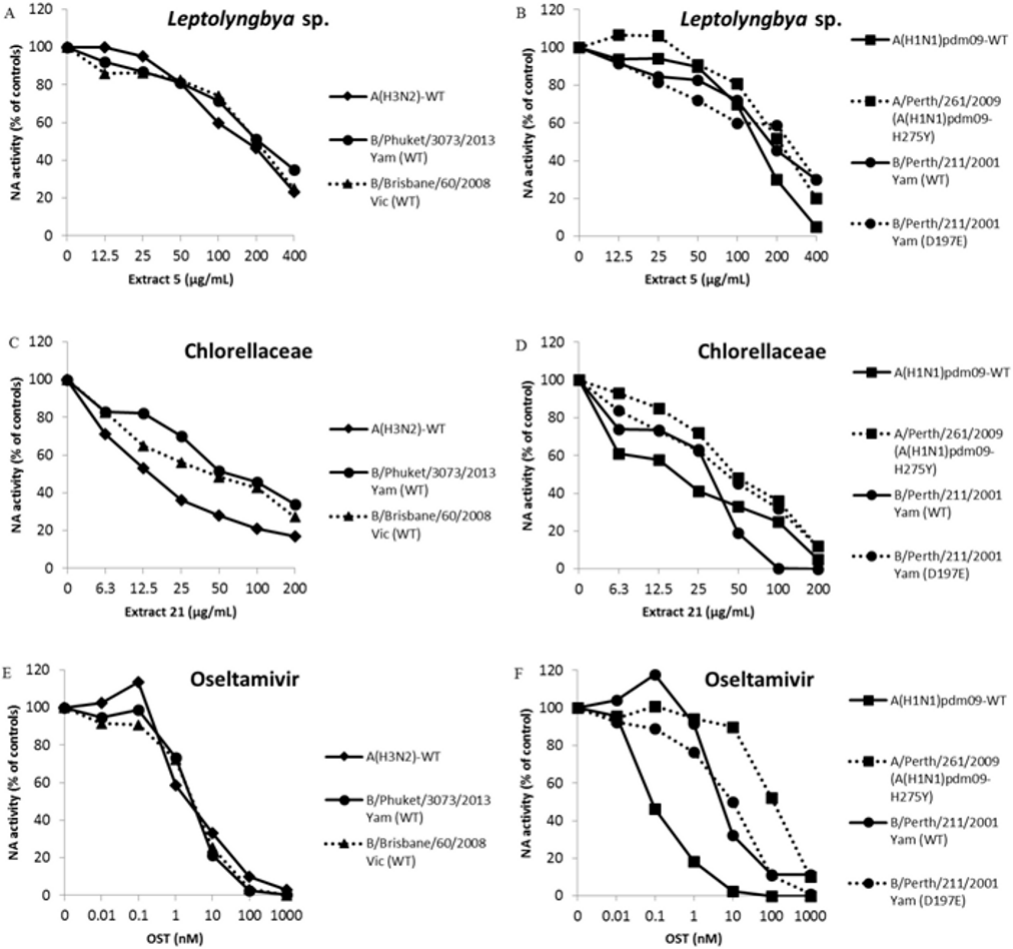
*Leptolyngbya sp*. and Chlorellaceae extracts inhibit influenza A and B OST-sensitive and -resistant viruses. The NA activity of the influenza A and B OST-sensitive and -resistant viruses were measured in the presence of different concentrations of extracts using a chemiluminescent substrate, NA-star kit (Life Technologies, Carlsbad, CA, USA). (A and B) Extract 5 (400 − 12.5 µg/mL). (C and D) Extract 21 (200 − 6.25 µg/mL). (E and F) OST carboxylate (1,000 − 0.01 nM), the reference compound was used as control. The results were obtained in relative luminescence units (RLU), but were converted to a percentage of the control for normalization of the data displayed. These experiments were performed three times and the means were expressed in the graphs.

We performed a functional assay to analyze neuraminidase activity with all the seven extracts that inhibited influenza infection in more than 80%. IC50 is the concentration required to inhibit 50% of the influenza NA enzymatic activity. Thus, lower IC50 values imply in higher NA inhibition activity. Two of them, extracts from the cyanobacteria *Leptolyngbya* sp. (5) and microalga Chlorellaceae (21) inhibited influenza A NA activity with IC_50_ lower than 210 and 20 µg/mL, respectively (Table 4 and Figs. 1A–1D). The OST-car was used as control (Table 4 and Figs. 1E and 1F). The HPLC and mass spectrum of extracts 5 and 21 are shown *S01-1* and *S01-2*, respectively. The mass spectrum of extract 5 (*S01-3*) indicated the presence of chlorinated compounds m/z = 214.9850 and 232.9946. On the other hand, the mass spectrum of extract 21 (*S01-4*) exhibited signals that indicated the presence of peptides, terpenes and/or alkaloids.

**Table 4:** The EC_50_ and IC_50_ values from extracts for OST-sensitive and resistant influenza A and B viruses.

**Influenza Virus strain**	**EC**_50_^a^**(µg/mL)**		**IC**_50_^b^**(µg/mL)**		
	***Leptolyngbya*sp.(extract 5)**	**Chlorellaceae (extract 21)**	***Leptolyngbya*sp.(extract 5)**	**Chlorellaceae (extract 21)**	**OST-car (nM)**
A(H1N1)pdm09-WT	80	30	149 ± 5	16 ± 2	0.1 ± 0.2
A(H3N2)-WT	85	40	162 ± 3	14 ± 3	2.1 ± 0.3
A/Perth/261/2009 (A(H1N1)pdm09H275Y)	168	50	208 ± 4	72 ± 3	112 ± 2
B/Phuket/3073/2013 Yam (WT)	215	70	207 ± 5	68 ± 2	2.8 ± 1
B/Brisbane/60/2008 Vic (WT)	230	75	199 ± 4	41 ± 2	3 ± 0.5
B/Perth/211/2001 Yam (WT)	200	100	179 ± 3	30 ± 5	5.0 ± 1
B/Perth/211/2001 Yam (D197E)	260	120	184 ± 5	42 ± 4	10 ± 2

**Notes.**

a EC_50_, the concentration required to reduced inhibition of viral infection-induced cytopathogenicity by 50%. Values represent the mean of duplicate samples from three independent experiments.

b IC_50_, the concentration required to inhibit 50% of the NA enzymatic activity. Values represent the mean of duplicate samples from three independent experiments ± standard error.

OST-car OST carboxylate

### EC_50_ and IC_50_ determination to influenza A and B OST-sensitive and resistant viruses

The OST is the main antiviral used in clinics against Influenza A and B. The pressure imposed by this drug has led to the selection of resistant mutants (Dixit et al., 2013; Lopes e Souza et al., 2015; Souza et al., 2011). Thus, the search for new anti-influenza compounds that inhibits both types of influenza and their resistant strains or lineages is extremely important. Therefore, we tested extracts from *Leptolyngbya* sp. (5) and Chlorellaceae (21) against replication and NA activity of influenza A OST-resistant (Table 4 and Figs. 1B and 1D) and B OST-sensitive and resistant (Table 4 and Figs. 1A–1D) viruses. The OST-car was used as control (Table 4 and Figs. 1E and 1F). Both extracts inhibited influenza replication and NA activity with low EC_50_ and IC_50_, including for OST-resistant strains and lineages (Table 4 and Fig. 1). The raw data of the IC_50_ calculations are provided in *S02*.

## Discussion

Influenza virus is the most important pathogen that causes acute lower respiratory infections (WHO, 2018b). The OST, a neuraminidase inhibitor, is the main anti-influenza used in clinics, but strains resistant to this molecule have been in clinical treatments reported worldwide, mandating the development of novel therapeutics (Dixit et al., 2013; Lopes e Souza et al., 2015; Souza et al., 2011).

Microalgae and cyanobacteria are huge natural sources of compounds of high nutritional and medicinal value. These marine and freshwater organisms are a rich source of bioactive compounds such as vitamins, proteins with essential amino acids, polysaccharides, fatty acids, minerals, photosynthetic pigments, enzymes, and fiber (Gogineni & Hamann, 2018; Montalvao et al., 2016).

Cyanobacteria are a promising yet underexplored source for novel natural products, including antiviral compounds. More detailed screening studies have identified antiviral activities on sulfoglycolipids and lectins (Lopes et al., 2011; Mundt et al., 1997; Niedermeyer, 2015; Sharaf et al., 2010; Zainuddin et al., 2002). However, chemically different groups of compounds in cyanobacteria include alkaloids, lipopeptides, macrolides, and others (Kiuru et al., 2014). Several studies have been conducted to test microalgae compounds against pathogenic human viruses. Antiviral compounds extracted from microalgae are mainly polysaccharides. Polyunsaturated aldehydes, carotenoid, terpenes, and alkaloids are other bioactive molecules produced by microalgae (Falaise et al., 2016; Martinez Andrade et al., 2018; Sathasivam & Ki, 2018).

Thus, we tested anti-influenza activity of extracts derived from microalgae and cyanobacteria collected from Paraguaçu River, Shallow Lake System of Lençois Maranhenses, Guanabara Bay estuary and Abrolhos coral reefs.

In screening assay we observed that seven extracts were able to inhibit seasonal influenza A replication viruses in more than 80%, five of them with SI higher than 10. Aqueous and methanolic extracts of cultured cyanobacteria of Microcystis and Spirulina genus inhibit influenza replication with SI comparable to ethyl acetate extract from *Leptolyngbya* sp. (cyanobacteria) (Chen et al., 2016; Zainuddin et al., 2002). Besides that, one sulfated polysaccharide purified from the marine microalga, *Gyrodinium impudium*, inhibits influenza A infection with SI higher than 200, but not influenza B (Kim et al., 2012).

In addition, *Trichodesmium erythraeum* (Cyanobacterial) aplysiatoxin-related compounds debromoaplysiatoxin and 3-methoxydebromoaplysiatoxin displayed anti-CHIKV effects at concentrations that resulted in minimal cytotoxicity (Gupta et al., 2014). Activity against influenza A has been found for the cyclic depsipeptides named ichthyopeptins A and B isolated from cyanobacterium *Microcystis ichthyolabe* (Zainuddin et al., 2007). *Nostoc ellipsosporum* (Cyanobacterial) lectin named Cyanovirin-N (CV-N) is a potent anti-influenza A as well as anti-influenza B. The CV-N bounds directly to the viruses, inactivating them (Singh et al., 2017). The green algae and land plants form a monophyletic lineage named chlorophytes, the first is an ancestor of the second (Delaux et al., 2015). Therefore, it is possible that they present similar primary and secondary metabolites (Cheynier et al., 2013; Iwai & Yokono, 2017). A broad spectrum of plant extracts inhibit the influenza replication, including extracts from ethyl acetate solvent, with SI comparable to that observed in extracts 2, 4, 18, 21 and 24, from green microalgae *Staurastrum* sp., *Scenedesmus* sp., *Desmodesmus armatus*, Chlorellaceae and Desmidiaceae, respectively (Hour et al., 2013; Wu et al., 2010).

The Chlorellaceae family, mainly *Chlorella* genus, produce a broad spectrum of carotenoids with different anti-angiogenic activities, cardioprotective, anti-cancer, anti-diabetic, anti-inflammatory, anti-oxidant and others (Sathasivam & Ki, 2018). Furthermore, retinoids, products of carotenoid metabolism, inhibit mumps virus replication preventing the infection of healthy cells by induction of a retinoid inducible gene I (RIG-I), retinoic acid receptor (RAR) and interferon (Soye et al., 2013). Interferon-induced transmembrane proteins (IFITMs) inhibit infection of diverse enveloped viruses, such as West Nile, dengue, Zika and influenza by blocking virus-endosome fusion (Brass et al., 2009; Desai et al., 2014; Mesquita et al., 2014; Savidis et al., 2016). Thus, it is possible that the Chlorellaceae (microalgal) extract leads to the increase of this cell restrictive protein, suggesting a mechanism of action for the influenza replication inhibitory effect.

In addition, two extracts from *Leptolyngbya* sp. (cyanobacteria) and Chlorellaceae family (microalgae) inhibited the replication and neuraminidase activity of influenza A and B viruses, including OST-sensitive and resistant strains and lineages. These results were similar to that observed for a water extract of *Taxodium distichum* (Cupressaceae family), which presented excellent efficacy against both influenza viruses, particularly OST-resistant clinical isolates and swine-origin influenza strains (Hsieh et al., 2016). These data indicate that microalgae and cyanobacteria are a promising source of anti-influenza natural products.

As mentioned before, there are several cellular factors described as restricting the replication of influenza virus (Villalon-Letelier et al., 2017). Furthermore, despite the inhibitory effect against neuraminidase activity, the extracts may have molecules capable of inhibiting different stages of the viral replicative cycle, and/or they can activate host cell restriction factors, but these effects will still be investigated. Thus, microalgae and cyanobacteria are important marine and freshwater microorganisms for bioprospecting in antiviral research. The microorganisms evaluated in this study were collected in Brazil, but they are not exclusively Brazilian. It may be relevant to compare the metabolites production of the same taxonomic groups collected in different parts of the world.

## Conclusions

In this study we tested anti-influenza activity of extracts derived by microalgae and cyanobacteria collected from Paraguaçu River, Shallow Lake System of Lençois Maranhenses, Guanabra Bay estuary and Abrolhos coral reefs. We found seven extracts, in ethyl acetate solvent, that inhibit seasonal influenza A and B replication in MDCK cells in more than 80%. Two extracts from *Leptolyngbya* sp. (cyanobacteria) and Chlorellaceae family (microalgae) inhibited influenza A and B replication and neuraminidase activity, from OST-sensitive and resistance strains and lineages. Thus, these organisms are important for bioprospecting in antiviral research.

##  Supplemental Information

10.7717/peerj.5716/supp-1 File S1 Chomatograms and Mass Spectra for extracts 5 and 21

10.7717/peerj.5716/supp-2 File S2 Raw data for the calculation of IC_50_

## Additional Information and Declarations

### Field Study Permissions

The following information was supplied relating to field study approvals (i.e., approving body and any reference numbers): The field experiments were approved by Ministério do Meio Ambiente (MMA), Instituto Chico Mendes de Conservação da Biodiversidade (ICMBio) and Sistema de Autorização e Informação em Biodiversidade (SISBIO), approval number: 35854-2. The access to genetic heritage was approved by Conselho Nacional de Desenvolvimento Científico e Tecnológico (CNPq), approval number: 010339/2014-0; and Sistema Nacional de Gestão do Patrimônio Genético e do Conhecimento Tradicional Associado (SisGen), approval number: A03D5C4.

### Data Availability

The following information was supplied regarding data availability: The raw data are provided in the Supplemental Files.
