# Supplementary material for: Inhibitory effect of microalgae and cyanobacteria extracts on influenza virus replication and neuraminidase activity
Source: PeerJ. 2018 Oct 26;6:e5716. doi: 10.7717/peerj.5716 (PMC6204821; doi:10.7717/peerj.5716)
Supplement: File S1 [file peerj-06-5716-s001.docx]

Supplementary file 01

**S01-1. The chromatogram of extract 5 (200 μg/mL).** Method: 5-100% ACN in 20 min, 100% ACN until 28 min as mobile phase, flow 1 mL/min, λ = 254 nm. Column Phenomenex Luna C-18, 5 µm, 4.6X250 mm. HPLC VARIAN, ProStar 230 Ternary Solvent Delivery Module, ProStar 335 Photodiode Array Detector.

**S01-2. The chromatogram of extract 21 (200 μg/mL).** Method: 5-100% ACN in 20 min, 100% ACN until 28 min as mobile phase, flow 1 mL/min, λ = 254 nm. Column Phenomenex Luna C-18, 5 µm, 4.6X250 mm. HPLC VARIAN, ProStar 230 Ternary Solvent Delivery Module, ProStar 335 Photodiode Array Detector.


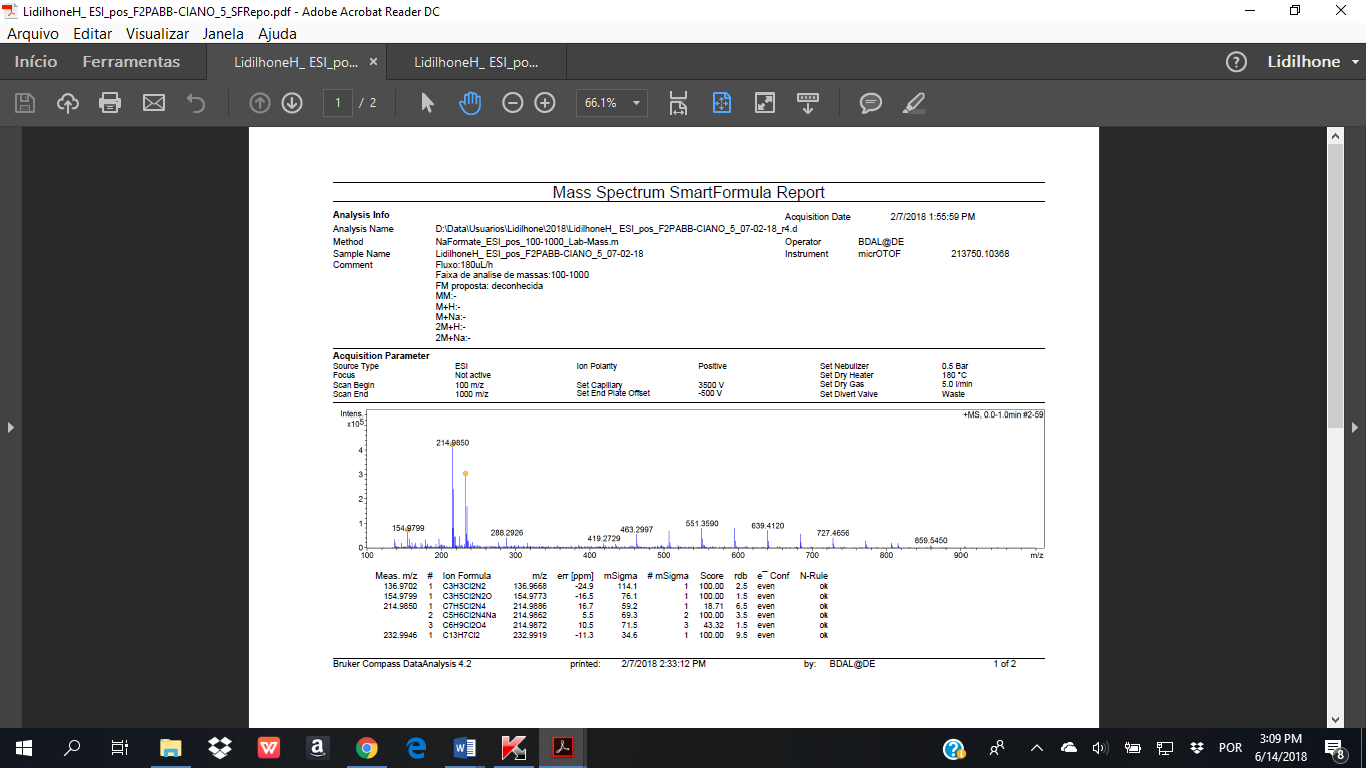


**S01-3. The mass spectrum of extract 5.**


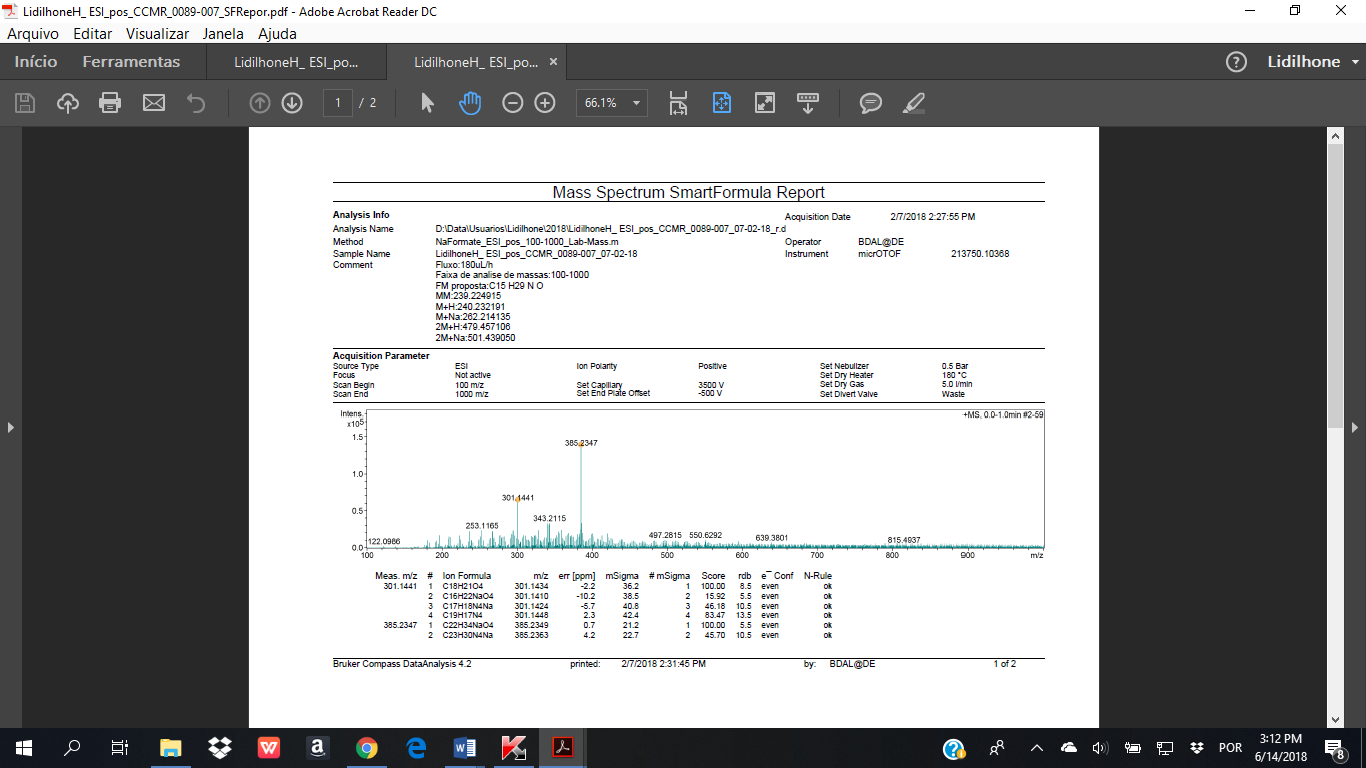


**S01-4. The mass spectrum of extract 21.**
